# Periodic Changes in the Gut Microbiome in Women with the Mixed Type of Irritable Bowel Syndrome

**DOI:** 10.3390/biomedicines13030652

**Published:** 2025-03-07

**Authors:** Aleksandra Kaczka, Aleksandra Błońska, Cezary Chojnacki, Anita Gąsiorowska, Janusz Błasiak, Tomasz Popławski, Jan Chojnacki

**Affiliations:** 1 Department of Clinical Nutrition and Gastroenterological Diagnostics, Medical University of Lodz, 90-647 Lodz, Poland; aleksandra.kaczka@umed.lodz.pl (A.K.); aleksandra.blonska@umed.lodz.pl (A.B.); cezary.chojnacki@umed.lodz.pl (C.C.); 2 Department of Gastroenterology, Medical University of Lodz, 92-213 Lodz, Poland; anita.gasiorowska@umed.lodz.pl; 3 Faculty of Medicine, Collegium Medicum, Mazovian Academy in Plock, 09-402 Plock, Poland; j.blasiak@mazowiecka.edu.pl; 4 Department of Pharmaceutical Microbiology and Biochemistry, Medical University of Lodz, 92-215 Lodz, Poland; tomasz.poplawski@umed.lodz.pl

**Keywords:** irritable bowel syndrome, gut microbiome, dysbiosis, breath tests, bacterial metabolites

## Abstract

**Background:** The mixed type of irritable bowel syndrome (IBS-M) is characterized by recurrent constipation and diarrhea. The cause of the variability of these symptoms is not sufficiently understood. The aim of this study was to perform metagenomic and metabolic assessment of the gut microbiome in constipation and diarrheal period of IBS-M. **Methods:** This study included 30 women, aged 28–47 years old, with the symptoms which aligned with those of IBS-M, according to the Rome IV Criteria. **Results:** In both periods of the disease, the dysbiosis index (DI), the Shannon diversity index (SDI), the hydrogen–methane and ammonia breath tests, as well as the selected bacterial metabolites (-p-hydroxyphenyl acetic acid (HPA), 3-indoxyl sulfate (Indican, 3-IS)), and hippuric acid (A) in urine, were determined. The dysbiosis index (DI) in the period of constipation was 3.73 ± 0.90 points, and in the diarrheal period it did not change significantly 3.93 ± 0.75 points (*p* > 0.05). During the diarrheal period, the diversity of bacteria increases from 2.16 ± 0.59 to 2.74 ± 0.50 points on the Shannon dietary index (*p* < 0.001). The gut microbiome profile also changed, especially during the diarrheal period where an abundance of *Bifidobacterium* spp. and *Lactobacillus* spp. decreased significantly. In addition, during this period, the levels of hydrogen and ammonia in breath air increased, while the methane level decreased. The differences also concern the results of urinary metabolites, especially related to hippuric acid and indican. During the diarrheal period, the levels of hydrogen and ammonia ions increased, while the methane level decreased. The differences also concern the results of urinary metabolites, especially related to hippuric acid and indican. **Conclusions:** In patients with IBS-M, periodic changes in the profile and metabolism of the gut microbiome occur, which coexist with recurrent symptoms such as constipation and diarrhea.

## 1. Introduction

Irritable bowel syndrome (IBS) is one of the most common diseases of the gastrointestinal tract, with a still unclear pathogenesis. Researchers are still looking for the causes of the ailments and their differentiation and recurrence. IBS manifests itself mainly in abdominal pain and bowel movement disorders. For this reason, the main forms of IBS with predominant constipation (IBS-C) and diarrhea (IBS-D) have been distinguished [1]. These symptoms are not permanent. It is considered that about half of patients may experience a subtype change over a period of several years [2]. In addition, in some patients these symptoms change alternately over the course of months [3,4]. This form of the disease has been referred to as mixed (IBS-M). One of the reasons for the variability of symptoms may be seasonal disturbances in the rhythm of secretion of biologically active compounds in the gastrointestinal tract. Particular attention has been paid to serotonin and dopamine, which are modulators of motor and secretory activity in this organ. About 90% of serotonin is secreted by enterochromaffin cells, but it is also secreted by neuronal and epithelial cells [5,6]. Similarly, about 50% of dopamine is synthesized in visceral neurons and non-neuronal cells in the intestinal walls [7,8]. Disturbances in the homeostasis of both neurotransmitters are the cause of functional changes in the gastrointestinal tract. Increased expression of tryptophan hydroxylase and an increase in serotonin secretion were found in patients with IBS-D [9,10]. In contrast, in the case of functional constipation, an increase in dopamine predominated [11,12]. The balance of the secretion of these neurotransmitters is influenced by many factors, including nutritional factors and gut microbiota [13]. In numerous studies, it has been shown that some bacteria have the ability to synthesize serotonin or dopamine and indirectly affect the pool of these neurotransmitters [14,15]. In our own previous studies, it has been shown that serotonin and dopamine levels depend on the type of IBS [16]. In another study, it was found that in patients with IBS-M the metabolism of tryptophan and the levels of bacterial metabolite change periodically [17]. Concentrations of these metabolites in the exhaled air, mainly hydrogen ions, were different during the constipation and diarrheal periods of this syndrome. The value of the hydrogen breath test is questioned by some researchers as hydrogen levels are also affected by other metabolites such as methane and hydrogen sulfide. In addition, food intake was not sufficiently monitored in these studies. This encouraged us to conduct further research in this area.

The aim of the study was to perform metagenomic and metabolic assessment of the gut microbiome in constipation and diarrheal period of IBS-M.

## 2. Material and Methods

### 2.1. Participants and Study Design

The study included 30 women, aged 28–47 years old, with constipation and diarrhea which allowed for diagnosis of the mixed type of irritable bowel syndrome (IBS-M). The diagnosis was consistent with the Rome IV Criteria [1], and the duration of the disease was from 3 to 8 years. The Gastrointestinal Symptom Rating Scale (GSRS-IBS) adopted for the European population [18] included the following symptoms: abdominal pain, pain relieved by bowel action, bloating, passing gas, constipation, diarrhea, loose stool, urgent bowel movement, incomplete intestinal emptying, fullness a short time or a long time after eating, and visible distension, which are scored between 1 and 7 points [18]. Recruitment of patients and the undertaking of the research was carried out at the Gastroenterology Clinic of the Central Hospital of the Medical University of Lodz. All patients experienced different bowel movements, in the form of constipation less than 3 times a week or loose/watery stools ranging from 3 to 8 per day.

The exclusion criteria were as follows: endoscopic and histological changes in gastric, duodenal, jejunal or colonic mucosa, celiac disease, allergy, food intolerance, liver and renal disease, diabetes and other metabolic disease, severe anxiety, and depression. These criteria also included the use of any drugs and contraceptives.

Medical and diagnostic tests, including metagenomic and metabolic assessment of the gut microbiota, were started during the constipation period and lasted at least 3 months. The patients were advised to maintain their current diet, which was monitored by a dietician. In addition, patients were required to fill out a daily diary detailing all their symptoms. During this time, the use of any medications was prohibited, except for soluble fiber (Mannodis GASTRO, Nerr Pharma, Łomianki Dolne, Poland). Further medical examinations were performed at 14 day intervals, and in the event of recurrence of diarrhea, in order to perform routine laboratory tests. Assessment of profile and microbial metabolism was performed in 3rd–4th week of the diarrhea phase after nutritional intervention.

### 2.2. Laboratory Tests

The laboratory tests included the following: blood count, glucose, glycated hemoglobin, lipids, bilirubin, iron, urea, creatinine, thyroid stimulating hormone, free thyroxine and triiodothyronine, antibodies to tissue transglutaminase, deaminated gliadin peptide, liver, kidneys and pancreas function indicators, C-reactive protein, fecal calprotectin, and bacterial metabolites in urine. Urine samples for indole compounds were collected in the morning, on an empty stomach, into a special container with a 0.1% hydrochloric acid solution as a stabilizer. A total of 12 organic acids were tested, including the following: -p-hydroxyphenylacetic acid (HPA), 3-indoxyl sulfate (Indican, 3-IS), and hippuric acid (HA). These metabolites were determined in the programs Organix Neuro and Organix Gastro (ALAB laboratories, Warsaw, Poland), using liquid chromatography with tandem mass spectrometry (LC–MS/MS, with Nexera fluorscent detection (Shimadzu, Kyoto, Japan, software-GCMS Solution version 4.50)). The levels of these metabolites were expressed in mg per gram of creatinine (mg/gCr). These results were considered as indirect indicators of the state of the intestinal microbiome.

### 2.3. Breathing Tests

Four weeks before the breath tests, the use of antibiotics and probiotics was prohibited, and a week before the consumption of easily fermentable ingredients was limited and fiber supplementation was discontinued. The hydrogen–methane breath test (HBT) was performed using a GastroCH_4_ECK Gastrolyzer (Gastro CHART^TM^ software, Bedfont, Ltd., Harrietsham, UK). The levels of hydrogen and methane in breath were measured at the initial time, and after administering 10 g of lactulose dissolved in 200 mL of water. Breath samples were collected at 20 min intervals for 3 h. The criterion was in accordance with the North American Breaths Testing Guideline [19]. However, the highest increase in hydrogen during the test was used for statistical evaluation, as an indicator of the microbiome status in the small intestine and colon.

The ammonia breath test (ABH) was performed using a gas analyzer (HELIC ABT Reader, AMA Co Ltd., Mikkeli, Finland). The concentration of ammonia (NH3) in the expiratory air was determined when the patients were fasting and then also at 20 min intervals for 3 h after ingestion of 250 mL of a protein solution (Nutridrink Protein—Nutricia).

Both tests, i.e., HBT and ABT, were performed in the same week, after discontinuing antibiotics, probiotics, and nutritional correction. The highest concentration increases in all of the above ions (hydrogen, methane, ammonia) was used for statistical analysis.

### 2.4. Dysbiosis Test

The GA-map Dysbiosis Test (GA-map^®^ Dysbiosis Test, Oslo, Norway), according to the method described by Casén et al. [20], was used to identify and characterize dysbiosis through differences between the study groups. A unique algorithm facilitates the determination of dysbiosis level and deviation from a normobiotic state in a clinical setting. The dysbiosis index (DI) has been established from 1 to 5, where a value of 2 or lower indicates the patient being non-dysbiotic, and a DI above 2 confirms dysbiosis, which is calculated using the specialized GA-map^®^ Analyzer Software v 1.4 (MAGPIX, Luminex, Austin, TX, USA). This method uses 54 bacterial markers based on the 16S rRNA sequences in 7 variable regions (V3–V9) and measures the relative abundance of bacteria. Each bacterial DNA marker is a source of different bacterial groups or species, which allows detection of over 300 bacteria from various taxonomic categories. In our study, the following phylum of bacteria were tested: Actinomycetota, Bacteroidota, Bacillota, Pseunomanadota, Mycoplasmatota, and Verrucomicrobiota, using the current nomenclature (FloraGen test, ALAB Laboratories, Warsaw, Poland).

### 2.5. Nutritional Recommendations

In the constipation period of IBS-M, the participants were recommended to maintain their current balanced diet of a total caloric value of 2000 kcal, and with a daily intake of a minimum of 50 g of protein, 270 g of carbohydrates, 70 g of fats, and 40 g of soluble fiber. One week before the breath and metabolic tests, phenylalanine and tryptophan were limited to a daily intake of 40 mg, and 15 mg per kilogram of body weight, respectively. Patients from both groups were recommended to complete a diet diary daily, under the control of competent dietitians, with whom they had telephone and e-mail contact. The average of their consumption was calculated using the nutritional calculator with the Kcalmar.pro premium application (Hermex, Lublin, Poland).

### 2.6. Ethical Issues

This research was conducted as an open-label clinical trial. Written consent was obtained from all participants. The study was conducted according to the guidelines of the Declaration of Helsinki and the Guidelines for Good Clinical Practice and approved by the Bioethics Committee of the Medical University of Lodz (RNN/176/18/KE).

### 2.7. Data Analysis

Normality of data distribution was checked using the Shapiro–Wilk W test. To analyze the differences between two paired groups, we used Student’s *t*-test, whereas for two unpaired groups of laboratory results, we used the Mann–Whitney U test. The two-proportion z test was used for comparing the percentage differences in the occurrence of various bacteria in different periods of irritable bowel syndrome. The correlations between the dysbiosis index and the results of hydrogen, methane, and ammonia levels, as well as urine metabolites, were analyzed using the Spearman rank test with the rho rank correlation coefficient (r). Differences were considered significant at *p* < 0.0, which were adjusted for multiple comparison by Bonferroni. All statistical analyses were performed with STATISTICA 13.3 software (TIBCO Software Inc., Palo Alto, CA, USA).

## 3. Results

In this study group, the period with constipation lasted from 3 to 7 (mean 4.6) months, while the diarrheal period lasted 3–5 (mean 2.4) months.

Nutritional status and liver and kidney function parameters were similar in both disease periods. There were no significant changes in levels of female hormones, and the significant dispersion of their values resulted from testing at different phases of the menstrual cycle. A significant increase in calprotectin concentration in the diarrheic period of IBS-M was observed, but they did not exceed the laboratory standard (Table 1).

None of the patients had a normal microbiome profile compared to the reference group. In 26 patients with constipation and 27 patients in the diarrheal period, the study revealed a high dysbiosis index (DI > 2 points, *p* > 0.05). In the constipation period, several taxonomic differences were detected, including increased relative abundance of *Bifidobacterium* spp. (13 people), *Lactobacillus* spp. (12), and Firmicutes Varia (9), (Table 2). These proportions changed during the diarrheal period. Namely, the increased abundance of *Bifidobacterium* spp. was confirmed in 8 patients and of *Escherichia* spp. in 10 patients, while the number of patients with increasing abundance of *Bifidobacterium* and *Lactobacillus* spp. decreased significantly. In addition, the number of patients with high levels of *Clostridium* spp. and *Streptococcus* spp. increased insignificantly (Table 2). Both the increase and decrease in the number of the other bacteria were insignificant.

**Figure 1:**
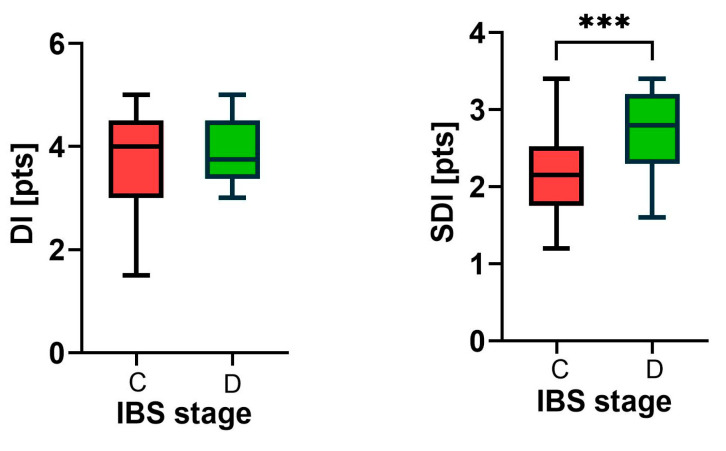
Dysbiosis index (DI) and Shannon diversity index (SDI) in mixed-type irritable bowel syndrome (IBS) patients during the constipation (C, red) and diarrheal (D, green) stages are presented as medians with minimum and maximum values, *n* = 30. Differences between stages were assessed using the Mann–Whitney U test. Pts—points, *** *p* < 0.001.

The dysbiosis index (DI) in the constipation period was 3.73 ± 0.90 points, and in the diarrhea period it had not changed significantly 3.93 ± 0.75 points (*p* > 0.05). During the diarrhea period, the diversity of bacteria increased from 2.16 ± 0.59 to 2.74 ± 0.50 points on the Shannon dietary index (*p* < 0.001, Figure 1).

**Figure 2:**
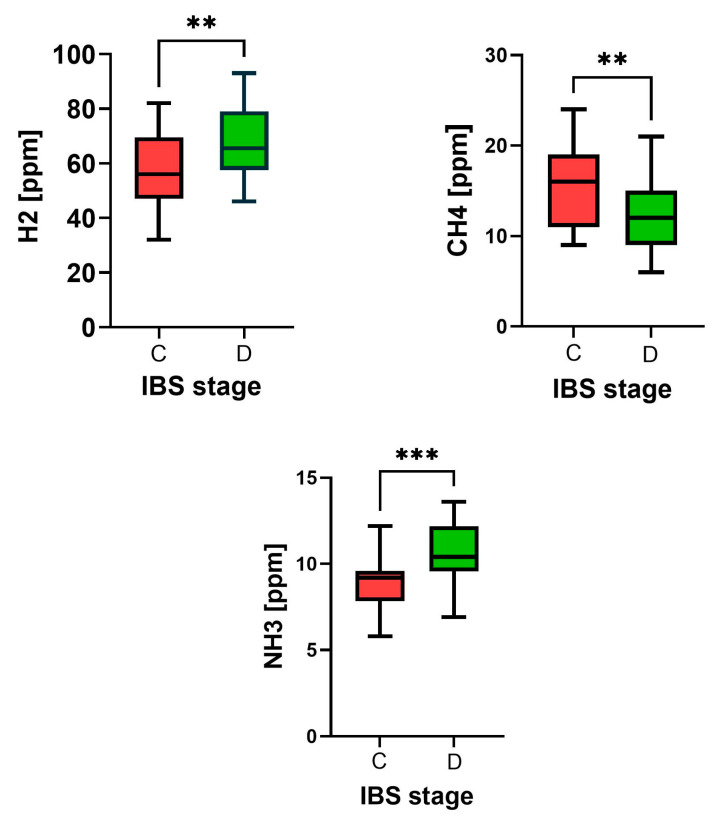
The highest concentrations (ppm) of hydrogen (H2), methane (CH4), and ammonia (NH3) in exhaled air were determined through appropriate breath tests conducted on mixed irritable bowel syndrome (IBS) patients during both the constipation (C, red) and diarrhea (D, green) stages. The median, along with minimum to maximum values, is *n* = 30, and differences between groups were assessed using the Mann–Whitney U test, **—*p* < 0.01, ***—*p* < 0.001.

Differences in the results of breathing tests were also found. During the diarrheal period, the levels of hydrogen and ammonia ions increased from 57.6 ± 12.9 to 68.5 ppm (*p* = 0.0024), and from 9.43 ± 2.81 to 10.55 ± 1.69 ppm (*p* = 0.0012), while the methane level decreased from 15.7 ± 4.63 to 12.1 ± 3.76 ppm (*p* = 0.0016, Figure 2).

The differences also concern the results of urinary metabolites. The levels of hippuric acid and indican levels decreased: 888.3 ± 82.4 vs. 798.5 ± 81.6 mg/gCr (*p* < 0.001) and 84.3 ± 12.4 vs. 71.5 ± 9.16 mg/gCr (*p* < 0.001), respectively, while the level of p-hydroxyphenyl acetic acid did not change significantly 24.8 ± 8. 3 vs. 26.2 ± 8.45 mg/gCr (*p* = 0.5412).

During the period of constipation, a positive correlation between the dysbiosis index and the methane level and a negative correlation with the hydrogen level in the exhaled air was demonstrated. However, during the diarrheal period, there was only a correlation between the DI and hydrogen levels. Such correlations were not found for urinary metabolites in the period of constipation (Table 3).

The cooperation of patients in the implementation of the research program was good. In particular, the adherence to the diet did not raise any objections from the dietitians. All patients completed the study within the applicable time frame.

## 4. Discussion

The results obtained confirm the participation of intestinal bacteria in the pathogenesis of IBS-M. In the study group of patients, the dysbiosis index was significantly higher compared to the reference group of healthy controls, but did not depend on the change in the IBS period. On the other hand, during the diarrhea period, the value of the diversity index increased. The reasons for these differences are not clear. Despite monitoring the diet of patients, differences in the consumption of the quantity and quality of products in the summer, autumn, and winter seasons cannot be ruled out. The composition of the microbiome is also influenced by the physical activity of patients during these periods. On hot summer days, viral infections that disrupt the intestinal ecosystem are also more common. Changes in the gut microbiome in IBS have been found by many researchers, but the results obtained were not consistent. Pittayanon et al., [21] on the basis of analysis of 24 studies, have shown that in this syndrome the overgrowth of bacteria from the Enterobacteriaceae and Bacteroides families is most common, while the number of *Bifidobacteriacae* and *Faecalibacterium* decreases. Jeffery et al. [22] in another meta-analysis showed a reduction in *Bifidobacterium, Lactobacillus*, and *Faecalibacterium prausnitzi*, whereas Duan et al. [23] found an increase in Firmicutes levels and a decrease in Bacteroides. The analysis by Wang et al. [24] shows that in people with IBS, the number of Escherichia coli and Enterobacter increases, and the number of *Lactobacillus and Bifidobacterium* decreases. In these studies, IBS types and methods of microbiome assessment were not distinguished. Other studies have found that in patients with IBS-D, compared to healthy people, bacteria from *the Bacteroidetes, Firmicutes*, and *Enterobacteriacae* predominate [25,26], and the number of *Lactobacillus* is decreased [24,27]. Liu, [28] in a review with a meta-analysis of 13 publications, showed differences in the microbiome profile in people with IBS-D, which specifically affected *Bifidobacterium, Lactobacllus*, and *Faecalibacterium praustnitzi*, but such differences were not observed in the IBS-C group [29]. Other researchers showed higher levels of *Firmicutes, Bacteroidetes*, and *Bifidobacterium* in people with IBS-C, and lower levels of *Actinobacteria and Bacteroidetes* [20,21,22,23,24,25,26,27,28,29,30,31,32]. Often, studies have focused on microbiome changes in patients with functional constipation, who have been found to have an increase in the number of bacteria such as *Bacteroidetes, Bifidobacterium, Clostridia*, and *Firmicutes* [33], or a decrease in the number of bacteria from the *Bifidobacterium, Bacteroidetes, Clostridium,* and *Lactobacillus* families [32,33,34,35].

The large discrepancy in results can have several causes, including dietary diversity. In our research, the reference group is people who eat Western and Mediterranean diets. The differences in these diets are in macro- and micronutrients, including animal and plant protein, as substrates to produce many bioactive compounds in the gastrointestinal tract. Secondarily, the differences may concern the microbiome profile, but in the Polish population they have not been confirmed in relation to IBS-C and IBS-D [36]. In these gut microbiome studies, various methods were used and a variety of bacterial strains that are rarely found in the gastrointestinal tract were determined. In our own research, the bacterial genes most often colonizing the intestines in the European population were isolated, including those from the Bacteroidetes and Firmicutes families, which constitute the relative majority of the microbiome, but its significant variability was demonstrated in the study group of patients. During the constipation period, dysbiosis was associated with a change in bacteria, particularly *Lactobacillus, Bifidobacterium,* and *Firmicutes*. These proportions changed during the diarrhea period, namely there was an increase in the number of patients with *Biidobacterium*, *Escherichia coli*, and *Clostridium* spp. overgrowth, while the number of patients with reduced numbers of *Fecalibacterium praustnitzi* and *Lactobacillus* decreased. These changes occurred over a period of 4 to 8 months. The dynamics of changes in the intestinal microbiome, especially under the influence of several weeks of probiotic therapy, have been pointed out by other researchers [37,38,39]. In the current study, some researchers point to very frequent changes in the symptoms and the results of breath tests in patients with SIBO [36]. Even daily measurements of hydrogen and methane in the exhaust air did not give identical results. Nevertheless, the above studies confirm the usefulness of breath tests in clinical practice. A particular modified method with simultaneous determination of hydrogen, methane, and hydrogen sulfide ions increases the value of these tests [40,41]. In our material, the results of the hydrogen–methane test correlated with the dysbiosis index, which provides additional information about the profile of the gut microbiome. The ammonia test is less commonly performed, but its results indicate levels of bacteria such as *Yersinia enterocolitica*, *Proteus* spp. and others [42,43].

In addition to breath tests, the level of bacterial metabolites in serum or urine are also considered as indirect indicators of dysbiosis determined. These metabolites, in addition to gases (H_2_, CH_4_, H_2_S, CO_2_, NO, and NH_3_), include short-chin fatty acids, bile acids, tryptophan and indole derivatives, and others [44,45,46]. Currently, in addition to profile (metagene) dysbiosis, the term metabolic dysbiosis is also recognized [47]. In our research, the concentrations of p-hydroxyphenyl acetic, 3-indoxyl sulfate, and hippuric acid, which are metabolites of phenylalanine, L-tryptophan, and aromatic compounds, respectively, were investigated. All these metabolites are considered as an indicator of bacterial overgrowth and the putrefaction process of protein and carbohydrate products. The correlations between the dysbiosis index and these metabolites were not found, but the results of other researchers indicate the advisability of making them [48,49]. However, correlations were demonstrated between DI and the level of hydrogen and methane ions, which confirmed their usefulness in the diagnosis of dysbiosis.

Regarding the health of patients, the results obtained have therapeutic implications and point toward eubiosis. The basis of treatment should be a proper diet, with monitoring of macro- and micronutrients in its composition. Dietary treatment should be modified based on the results of gut microbiome research, using various methods. These results can also be used in the selection of an appropriate probiotic for the treatment of gut dysbiosis.

Our research has some limitations. Firstly, the studied group of patients is relatively small and consists only of women. Secondly, the effect of female hormones and the phase of the menstrual cycle on the results obtained has not been studied. Thirdly, some types of bacteria are closely related and difficult to distinguish using 16S rRNA sequence-based methods.

Many studies have shown the critical role of the gut microbiome in the pathogenesis of IBS [50,51]. The results of our research confirm that IBS-M patients experience changes in the gut microbiome profile and in the metabolism of intestinal bacteria [52]. As a result, the bacterial metabolism of food products changes, which may be the cause of the diversity and variability of clinical symptoms of this disease. Reports on the effectiveness of probiotics (usually *Bifidobacterium* and *Lactobacillus*) in IBS-M patients are rare, and the results are questionable. In a recent study, Matsuura et al. [53] showed good results in the treatment with the above probiotics in IBS-D and IBS-C patients, but not with IBS-M. Finally, metagenomic, metabolomic, and clinical research for the personalized treatment are necessary in the future.

## 5. Conclusions

In patients with IBS-M, periodic changes in the profile and metabolism of the gut microbiome occur, which coexist with recurrent symptoms such as constipation and diarrhea.

## Figures and Tables

**Table 1:** General characteristics and the selected laboratory data in women (*n* = 30) with the mixed type of irritable bowel syndrome (IBS-M) in constipation and diarrheal period.

Feature	Constipation Period	Diarrheal Period	*p*-Value
Age (years)	38.1 ± 6.6	38.3 ± 7.1	0.869
BMI (kg/m^2^)	23.2 ± 0.9	23.0. ± 1.1	0.067
GFR (mL/min)	99.1 ± 4.9	97.9 ± 5.5	0.061
ALT (µ/L)	15.6 ± 3.5	14.9 ± 3.8	0.059
AST (µ/L)	13.9 ± 2.1	14.3 ± 1.8	0.092
CRP (mg/L)	2.3 ± 1.8	3.1 ± 2.3	0.188
FC (µg/g)	26.6 ± 15.7	30.9 ± 12.8	0.045 *
ES (pg/mL)	118.6 ± 36.2	136.8 ± 41.1	0.068
FSH (IU/L)	12.1 ± 10.5	14.9 ± 12.9	0.089
TSH (µIU/L)	2.12 ± 0.58	3.12 ± 0.83	0.079

BMI—body mass index; GFR—glomerular filtration rate; ALT—alanine aminotransferase; AST—aspartate aminotransferase, CRP—C-reactive protein; FC—fecal calprotectin; ES—17-β-estradiol; FSH—follicular-stimulating hormone; TSH—thyroid-stimulating hormone. Differences between groups were assessed by Student’s *t*-test; *—*p* < 0.05.

**Table 2:** Number and percentage (No/%) of IBS-M patients with an increased abundance (2 and 3 score) of bacteria in the constipation (C) and diarrhea (D) period; differences were assessed using the two proportions z-test. * *p* < 0.05.

Bacteria Species	Period-C (No/%)	Period-D (No/%)	*p*-Value
*Bacteroides*	3 (10.0)	8 (26.6)	0.1068
*Bifidobacterium* spp.	13 (43.3)	8 (26.6)	0.0496 *
*Clostridium* spp.	3 (10.0)	6 (20.0)	0.2781
*Escherichia* spp.	6 (20.0)	10 (33.3)	0.2539
*Faecalibacterium prausn.*	7(23.3)	3 (10.0)	0.1750
*Furmicutes* (varia)	9 (30.0)	7 (23.3)	0.5390
*Lactobacillus* spp.	12 (40.0)	4 (13.3)	0.0178 *
*Proteobacteria* spp.	4 (13.3)	8 (26.6)	0.1974
*Ruminococcus* (varia)	4 (13.3)	6 (20.0)	0.4861
*Streptococcus* spp.	3 (10.0)	6 (20.0)	0.2781

**Table 3:** Correlation between the dysbiosis index (DI) and the level of tested metabolites: H2–hydrogen; CH4–methane; NH3–ammonia; HPA–p-hydroxyphenyl acetic acid; 3-IS–3-indoxyl sulfate; HA–hippuric acid. The correlations were analyzed using the Spearman rank test with the rho rank correlation coefficient (r); * *p* were adjusted for multiple comparison by Bonferroni. * *p* < 0.05 ** *p* <0.01, *** *p* < 0.001.

DI vs. Urine Metabolites	Rho Spearman/p			
	Period-C	*p*-Value	Period-D	*p*-Value
H2	−0.9598	0.0089 **	0.8425	0.0006 ***
CH4	0.6776	0.0360 *	−0.2466	0.2570
NH3	0.3796	0.2675	0.1275	0.4975
HPA	−0.0313	0.0783	−0.0398	0.8467
3-IS	0.1504	0.1504	0.0266	0.8946
HA	−0. 1807	0.9998	0.0647	0.7723

## Data Availability

Data from this study are ready to be shared upon reasonable request.
